# RNMFMDA: A Microbe-Disease Association Identification Method Based on Reliable Negative Sample Selection and Logistic Matrix Factorization With Neighborhood Regularization

**DOI:** 10.3389/fmicb.2020.592430

**Published:** 2020-10-27

**Authors:** Lihong Peng, Ling Shen, Longjie Liao, Guangyi Liu, Liqian Zhou

**Affiliations:** School of Computer Science, Hunan University of Technology, Zhuzhou, China

**Keywords:** microbe-disease associations, reliable negative samples, positive-unlabeled learning, random walk with restart, logistic matrix factorization with neighborhood regularization

## Abstract

Microbes with abnormal levels have important impacts on the formation and development of various complex diseases. Identifying possible Microbe-Disease Associations (MDAs) helps to understand the mechanisms of complex diseases. However, experimental methods for MDA identification are costly and time-consuming. In this study, a new computational model, RNMFMDA, was developed to find possible MDAs. RNMFMDA contains two main processes. First, Reliable Negative MDA samples were selected based on Positive-Unlabeled (PU) learning and random walk with restart on the heterogeneous microbe-disease network. Second, Logistic Matrix Factorization with Neighborhood Regularization (LMFNR) was developed to compute the association probabilities for all microbe-disease pairs. To evaluate the performance of the proposed RNMFMDA method, we compared RNMFMDA with five state-of-the-art MDA prediction methods based on five-fold cross-validations on microbes, diseases, and MDAs. As a result, RNMFMDA obtained the best AUCs of 0.6332, 0.8669, and 0.9081, respectively for the three five-fold cross validations, significantly outperforming other models. The promising prediction performance may be attributed to the following three features: highly quality negative MDA sample selection, LMFNR-based MDA prediction model, and various biological information integration. In addition, a few predicted microbe-disease pairs with high association scores are worthy of further experimental validation.

## 1. Introduction

Microbes are the most abundant microscopic organisms on Earth and control many major biological and chemical processes (Ley et al., 2006; Qu J. et al., 2019; Sachdeva et al., 2019). Normal microbial flora are beneficial for the host health (McFarland, 2000; Langella and Mart́ın, 2019; Qu J. et al., 2019). Beneficial microbes including biotherapeutic agent, probiotics and synbiotics have been reported as effective therapeutic clues when normal microflora are disrupted (McFarland, 2000; Langella and Mart́ın, 2019).

More importantly, microorganisms have an important affect on infectious diseases and non-infectious diseases (Findley et al., 2013; Ding and Schloss, 2014; Abu-Ali et al., 2018; Byrd et al., 2018; Liu et al., 2019). The human body is possible to get sick when foreign microorganisms invade or a microbial community is imbalanced (Zhu et al., 2018; Qu K. et al., 2019). For example, there are more abundant *Fusobacterium* in asthmatic patients than healthy people (Davis-Richardson et al., 2014). Lecithinase-negative *Clostridium* and *Lactobacillus* are much more in colorectal carcinoma patients (Heavey and Rowland, 2004). Increased *Lactobacillus* can result in tertiary lymphoid (Sze et al., 2012). All the above reports suggested that there are close associations between microbes and human diseases. Therefore, finding new Microbe-Disease Associations (MDAs) helps to provide diagnostic and therapeutic clues for clinical researches Chen et al. (2017).

Experimental methods to predict possible MDAs are costly and time-consuming. Computational methods are thus gradually developed to find potential MDAs. Ma et al. (2016) collected published MDA data from literatures and constructed Human Microbe-Disease Association Database (HMDAD). Various computational models are then exploited based on known MDA data, Gaussian Interaction Profile Kernel (GIP) similarity for diseases and microbes. Chen et al. (2017) assumed that functionally similar microbes are likely to associate with similar non-infectious diseases and presented the first tool (KATZHMDA) to predict potential MDAs based on the KATZ measure. Huang et al. (2017) proposed a neighbor and graph-based recommendation model (NGRHMDA). Bao et al. (2017) designed a Network Consistency Projection-based MDA prediction method (NCPHMDA). Luo and Long (2018) constructed a heterogeneous network and presented a Network Topological Similarity-based human MDA prediction model (NTSHMDA). Wang et al. (2017) developed a semi-supervised learning framework (LRLSHMDA) to prioritize microbe candidates for all interested diseases based on Laplacian Regularized Least Squares. Peng et al. (2018b) exploited a adaptive boosting-based method to compute association scores for human microbe-disease pairs based on a strong classification model. Zhang et al. (2018) proposed a bi-direction similarity integration label propagation method (BDSILP) for identifying MDAs. Shi et al. (2018) assumed that observed incomplete microbe-non-infectious disease association matrix is composed of a parameterized matrix and a noise matrix, and then developed a Binary Matrix Completion-based model (BMCMDA) to infer possible microbe-non-infectious disease associations. Qu J. et al. (2019) presented a human MDA model (MDLPHMDA) based on matrix decomposition and label propagation.

The above methods were effectively applied to MDA identification and captured a few MDAs, however, the prediction performance remains to be improved. More importantly, in MDA identification problem, negative training examples are missing. Therefore, most of models randomly extracted negative MDAs from unknown microbe-disease pairs, which may contain positive MDAs, thereby severely affecting the prediction accuracy. Learning from Positive and Unlabeled examples (PU learning) (Li et al., 2014) is one type of methods used to learn the models from numerous positive and unlabeled examples. PU learning has been widely applied to text mining and obtained better performance.

In this study, we developed a computational model, RNMFMDA, to predict human MDA candidates. RNMFMDA integrated Reliable Negative MDA selection based on PU learning and random walk with restart, Logistic Matrix Factorization with Neighborhood Regularization (LMFNR), and multiple heterogeneous data. RNMFMDA first computed disease similarity and microbe similarity. Credible negative MDAs were then selected based on PU learning and random walk with restart. LMFNR was finally developed to identify MDA candidates. RNMFMDA was compared to five state-of-the-art MDA prediction methods, MDLPHMDA (Qu J. et al., 2019), NGRHMDA (Huang et al., 2017), NTSHMDA (Luo and Long, 2018), LRLSHMDA (Wang et al., 2017), and KATZHMDA (Chen et al., 2017). To evaluate our proposed RNMFMDA, we conducted five-fold Cross Validations (CVs) on microbes, diseases, and MDAs. The results showed that RNMFMDA obtained the best AUCs under the above three CVs. In addition, we further performed the experiments to find possible microbes/diseases associate with a known disease/microbe. The experimental result analysis suggested that RNMFMDA is a powerful MDA identification method.

## 2. Materials and Equipment

Assume that the *i*th microbe is represented as *m*_*i*_(*i* = 1, 2, …, *n*), and the *j*th disease is denoted as *d*_*j*_(*j* = 1, 2, …, *m*). The associations between *n* microbes and *m* diseases are denoted as a binary matrix ***Y***_(*n* × *m*)_ where

(1) $$\begin{array}{l} y_{ij}=\left\{ \begin{array}{c} \;1\;if\;m_{i}\;associates\;with\;d_{j}\\ 0\;otherwise \end{array}\right. \end{array}$$

The non-zero elements in ***Y*** are called “MDA pairs” and considered as positive observations. The zero elements in ***Y*** are called “unknown microbe-disease pairs” and considered as unlabeled observations. The microbe similarity matrix and the disease similarity matrix are represented as $S_{M}\in {ℜ}^{n\times n}$ and $S_{D}\in {ℜ}^{m\times m}$, respectively.

Our objective is to select reliable negative MDAs based on PU learning and random walk with restart on the heterogeneous network, and then compute the association probability score for each microbe-disease pair by LMFNR, finally rank candidate microbe-disease pairs according to the scores in descending order, so that the top microbe-disease pairs are the most likely to be MDAs.

We collected confirmed MDAs from HMDAD (Ma et al., 2016) (http://www.cuilab.cn/hmdad). The database provides 483 MDAs between 292 microbes and 39 diseases from 61 previous works. We deleted the same MDAs based on different evidences and finally obtained 450 MDAs from these microbes and diseases.

## 3. Methods

### 3.1. Microbe GAP Similarity

Motivated by the similarity computation method provided by van Laarhoven et al. (2011), we computed microbe Gaussian Association Profile (GAP) similarity based on known MDA matrix. Given a microbe *m*(*i*), its GAP ***AP***(*m*(*i*)) can be represented as the *i*th row of ***Y***. The GAP similarity between two microbes *m*(*i*) and *m*(*j*) can be computed by Equation (2):

(2) $$\begin{array}{l} S_{M}\left(m\left(i\right),m\left(j\right)\right)=\text{exp}\left(-\gamma_{m}\|AP\left(m\left(i\right)\right)-AP\left(m\left(j\right)\right){\|}^{2}\right) \end{array}$$

where $\gamma_{m}={\gamma_{m}}^{\prime }/(\frac{1}{n}\sum_{k=1}^{n}\left\|AP(m(k)){\|}^{2}\right)$ denotes the normalized kernel bandwidth with bandwidth parameter ${\gamma_{m}}^{\prime }$. The microbe similarity matrix ***S***_*M*(*n* × *n*)_ can be obtained based on the GAP similarity.

### 3.2. Disease Similarity

#### 3.2.1. Disease GAP Similarity

For a disease *d*(*i*), its GAP ***AP***(*d*(*i*)) can be represented as the *i*th column of ***Y***. The GAP similarity between two diseases *d*(*i*) and *d*(*j*) can be calculated by Equation (3):

(3) $$\begin{array}{l} S_{G}\left(d\left(i\right),d\left(j\right)\right)=\text{exp}\left(-\gamma_{d}\|AP\left(d\left(i\right)\right)-AP\left(d\left(j\right)\right){\|}^{2}\right) \end{array}$$

where $\gamma_{d}={\gamma_{d}}^{\prime }/(\frac{1}{m}\sum_{k=1}^{m}\left\|AP(d(k)){\|}^{2}\right)$ denotes the normalized kernel bandwidth with bandwidth parameter ${\gamma_{d}}^{\prime }$.

#### 3.2.2. Disease Symptom Similarity

Inspired by the similarity measure method provided by Zhou et al. (2014), we computed disease symptom similarity matrix ***S***_*S*_.

Finally, the disease similarity matrix ***S***_*D*(*m* × *m*)_ can be computed by Equation (4):

(4) $$\begin{array}{l} S_{D}\left(d\left(i\right),d\left(j\right)\right)=S_{G}\left(d\left(i\right),d\left(j\right)\right)+\gamma S_{S}\left(d\left(i\right),d\left(j\right)\right) \end{array}$$

where γ is a parameter used to weigh the importance between the GAP similarity and the symptom similarity.

### 3.3. Reliable Negative MDA Selection

There exists a few known MDAs and numerous unobserved microbe-disease pairs in the HMDAD database (Ma et al., 2016). There are no negative MDA samples because of the limitations of experimental methods. High-quality negative MDAs can boost the performance of MDA prediction models. Therefore, most of machine learning-based methods have to randomly select negative examples from unknown microbe-disease pairs. However, this part of randomly selected negative examples probably contains positive MDAs, thereby severely affecting the performance of MDA identification algorithms. Therefore, we developed a negative sample selection method to extract reliable negative MDA data based on PU learning and random walk with restart. The pipeline mainly contains two basic processes: computing the association probability for each microbe-disease pair based on random walk with restart and extracting high-quality negative MDA samples based on PU learning and the computed association scores.

#### 3.3.1. Random Walk With Restart on the Heterogeneous Microbe-Disease Network

Inspired by the method proposed by Chen et al. (2012), we consider microbe similarity network, disease similarity network, and MDA network to construct a heterogeneous microbe-disease network. We used microbe similarity matrix ***S***_*M*(*n* × *n*)_, disease similarity matrix ***S***_*D*(*m* × *m*)_, and MDA matrix ***Y***_(*n* × *m*)_ as the adjacency matrices of the above three networks, respectively. And the adjacency matrix on the heterogeneous network can be denoted as:

(5) $$\begin{array}{l} H=\left[\begin{array}{cc} S_{M} & Y\\ Y^{T} & S_{D} \end{array}\right] \end{array}$$

where ***Y***^*T*^ denotes the transpose of ***Y***.

We then calculate different transition probabilities of random walk with restart on the heterogeneous graph. Assume that $H=\left[\begin{array}{cc} H_{MM} & H_{MD}\\ H_{DM} & H_{DD} \end{array}\right]$ represent the transition probability matrix, where ***H***_*MM*_ and ***H***_*DD*_ represent the walks within microbe-microbe similarity network and disease-disease similarity network, respectively, ***H***_*MD*_ and ***H***_*DM*_ represent the skips between networks. Given a microbe/disease, if there exist a bipartite association between the microbe/disease and diseases/microbes, the particle will either skip between the four networks or stay in the current network with a transition probability λ ∈ [0, 1].

We predict MDA candidates from a perspective of microbes. Assume that a particle be situated on the *i*-th microbe node *m*_*i*_ ∈ *M*, it will walk to a microbe node *m*_*j*_ ∈ *M* with the transition probability ***H***_*MM*_(*i, j*):

(6) $$\begin{array}{l} H_{MM}\left(i,j\right)=\left\{ \begin{array}{cc} S_{M}\left(i,j\right)/\overset{n}{\underset{k=1}{\sum }}S_{M}\left(i,k\right), & \text{if}\overset{m}{\underset{k=1}{\sum }}Y\left(i,k\right)=0\\ \left(1-\lambda \right)S_{M}\left(i,j\right)/\overset{n}{\underset{k=1}{\sum }}S_{M}\left(i,k\right), & otherwise \end{array}\right. \end{array}$$

or skip to a disease *d*_*j*_ ∈ *D* based on a bipartite association with *d*_*j*_ with the transition probability ***H***_*MD*_(*i, j*):

(7) $$\begin{array}{l} H_{MD}\left(i,j\right)=\left\{ \begin{array}{c} \lambda Y\left(i,j\right)/\overset{m}{\underset{k=1}{\sum }}Y\left(i,k\right),\text{ if}\overset{m}{\underset{k=1}{\sum }}Y\left(i,k\right)\neq 0\\ \;0,\;otherwise \end{array}\right. \end{array}$$

Similarly, we can find possible MDAs from a perspective of diseases. Assume that a particle be situated on the *i*th disease node *d*_*i*_ ∈ *D*. It will walk to a disease node *d*_*j*_ ∈ *D* with the transition probability ***H***_*DD*_(*i, j*):

(8) $$\begin{array}{l} H_{DD}\left(i,j\right)=\left\{ \begin{array}{cc} S_{D}\left(i,j\right)/\overset{m}{\underset{k=1}{\sum }}S_{D}\left(i,k\right), & \text{if}\overset{n}{\underset{k=1}{\sum }}Y\left(k,i\right)=0\\ \left(1-\lambda \right)S_{D}\left(i,j\right)/\overset{m}{\underset{k=1}{\sum }}S_{D}\left(i,k\right), & otherwise \end{array}\right. \end{array}$$

or skip to a microbe *m*_*j*_ ∈ *M* based on a bipartite association with *m*_*j*_ with a transition probability ***H***_*DM*_(*i, j*):

(9) $$\begin{array}{l} H_{DM}\left(i,j\right)=\left\{ \begin{array}{c} \lambda Y\left(j,i\right)/\overset{n}{\underset{k=1}{\sum }}Y\left(k,i\right),\text{ if}\overset{n}{\underset{k=1}{\sum }}Y\left(k,i\right)\neq 0\\ \;0,\;otherwise \end{array}\right. \end{array}$$

Therefore, we describe random walk with restart on the heterogeneous network as:

(10) $$\begin{array}{l} P\left(t+1\right)=\left(1-\theta \right)H^{T}*P\left(t\right)+\theta P\left(0\right) \end{array}$$

where *P*(*t*) denotes a probability matrix used to represent the association scores of all unobserved microbe-disease pairs at the *t*-th step random walk, ***H***^*T*^ denotes the transpose of ***H***, and θ represents the restarting probability. The particle will return to either a seed microbe or a seed disease. More importantly, it is possible to differentiate the relative important of each network based on the initial probability $p_{i}(0)=\left[\begin{array}{c} (1-\eta )v_{i}\\ \eta s_{i} \end{array}\right]$, where *v*_*i*_ and *s*_*i*_ denote the initial probability distributions on disease-disease similarity network and microbe-microbe similarity network starting from their seed nodes, respectively. The parameter η ∈ [0, 1] is used to control the restarting probability in these two similarity networks. If η < 0.5, the particle will more tend to restart from one of the seed microbes than from one of the seed diseases.

#### 3.3.2. Reliable Negative MDA Extraction

We took known MDAs as initial positive sample set *P*, observed microbe-disease pairs as initial unlabeled sample set *U* and developed a reliable negative MDA selection based on PU learning. The method contains the following five steps:

Step 1. Randomly selecting positive sample subset *S* from *P* and adding *S* into *U*;
Step 2. Taking *P* − *S* as positive samples, *U* + *S* as negative samples;
Step 3. Computing the association score matrix ***AM*** based on random walk with restart on the heterogeneous microbe-disease network;
Step 4. Ranking microbe-disease pairs in *S* based on ***AM*** and finding the minimum score ***AM***_*min*_ in *S*;
Step 5. For every sample *x* in *U*:
                            if ***AM***_*x*_ satisfying ***AM***_*x*_ < ***AM***_*min*_
                            then *RN* = *RN* ∪ *x*

We can obtain reliable negative MDA example set *RN* with the above negative selection method.

### 3.4. MDA Prediction Based on LMFNR

The logistic matrix factorization method has widely applied to the area of various association prediction and obtained better performance (Liu et al., 2016, 2020). Inspired by the logistic matrix factorization method provided by Liu et al. (2016) and Liu et al. (2020), we developed an MDA prediction method (RNMFMDA) by integrating the Reliable Negative MDA sample selection method and the LMFNR method.

Suppose that both microbes and diseases are mapped into *r*-dimensional shared latent spaces where *r* ≪ *n, m*. The properties of a microbe *m*_*i*_ / disease *d*_*j*_ is represented by a latent vector $a_{i}\in {ℜ}^{1\times t}$ / $b_{i}\in {ℜ}^{1\times t}$. Then, the association probability *p*_*ij*_ between *m*_*i*_ and *d*_*j*_ can be computed by Equation (11):

(11) $$\begin{array}{l} p_{ij}=\frac{\text{exp}\left(a_{i}b_{j}^{T}\right)}{1+\text{exp}\left(a_{i}b_{j}^{T}\right)} \end{array}$$

The latent vectors of all microbes / diseases can be denoted as ***A*** ∈ ℜ^*n* × *r*^ / ***B*** ∈ ℜ^*m* × *r*^, where ***a***_*i*_ / ***b***_*j*_ is the *i*th / *j*th row in ***A***/***B***.

In MDA identification tasks, the observed MDAs have been experimentally validated and are more reliable than unknown microbe-disease pairs. To more accurately find MDA candidates, we assigned higher confidence scores to known MDAs than unknown pairs. Particularly, each MDA is considered as *c*(*c*≥ 1) positive training samples, and each reliable negative MDA is considered as a single negative training sample. *c* is a constant to measure the importance of observations. The importance weighting technique has been effectively applied to the area of informatics. And we built the following MDA prediction model:

(12) $$\begin{array}{l} \begin{array}{c} p\left(Y|A,B\right)=\left(\underset{1\leq i\leq n,1\leq j\leq m,y_{ij}=1}{\prod }{\left[p_{ij}^{y_{ij}}{\left(1-p_{ij}\right)}^{\left(1-y_{ij}\right)}\right]}^{c}\right)\\ \;\times \left(\underset{1\leq i\leq n,1\leq j\leq m,y_{ij}=0}{\prod }\left[p_{ij}^{y_{ij}}{\left(1-p_{ij}\right)}^{\left(1-y_{ij}\right)}\right]\right)\\ \;=\overset{n}{\underset{i=1}{\prod }}\overset{m}{\underset{j=1}{\prod }}p_{ij}^{cy_{ij}}{\left(1-p_{ij}\right)}^{\left(1-y_{ij}\right)} \end{array} \end{array}$$

The above model can represented as the following optimization function considering the probability distribution based on a Bayesian inference:

(13) $$\begin{array}{l} \begin{array}{c} \underset{A,B}{\min}\overset{m}{\underset{i=1}{\sum }}\overset{n}{\underset{j=1}{\sum }}\left(1+cy_{ij}-y_{ij}\right)\text{ log}\left[1+\text{exp}\left(a_{i}b_{j}^{T}\right)\right]\\ \;-cy_{ij}a_{i}b_{j}^{T}\;+\frac{\lambda_{m}}{2}\|A{\|}_{F}^{2}+\frac{\lambda_{d}}{2}\|B{\|}_{F}^{2} \end{array} \end{array}$$

where λ_*m*_ and λ_*d*_ are parameters, ||***A***||_*F*_ and ||***B***||_*F*_ denote the Frobenius norm of ***A*** and ***B***, respectively.

The nearest neighborhood information of biological entities in the association network can improve the prediction performance (Zhang et al., 2019a,b,c). For example, Zhang et al. (2019a), Zhang et al. (2019b), and Zhang et al. (2019c) used neighborhood information and effectively found microRNA-disease associations, drug-drug interactions and long non-coding RNA-miRNA interactions. Therefore, we integrated neighborhood information to the above optimization model and built the final LMFNR model by Equation (14):

(14) $$\begin{array}{l} \begin{array}{c} \underset{A,B}{\min}\overset{m}{\underset{i=1}{\sum }}\overset{n}{\underset{j=1}{\sum }}\left(1+cy_{ij}-y_{ij}\right)\text{ ln}\left[1+\text{exp}\left(a_{i}b_{j}^{T}\right)\right]-cy_{ij}a_{i}b_{j}^{T}\;\\ \;+\frac{1}{2}tr[A^{T}\left(\lambda_{m}I+\alpha L_{m}\right)A+\frac{1}{2}tr[B^{T}\left(\lambda_{d}I+\alpha L_{d}\right)B \end{array} \end{array}$$

where *tr*(·) denotes the trace of a matrix, ***L***_*m*_ and ***L***_*d*_ were defined as the same to Liu et al. (2016).

We can obtain ***A*** and ***B*** by solving with the optimization problem by Equation (14) with an alternating gradient ascent procedure.

Finally, the association probability matrix ***Y***_*p*_ for all unknown microbe-disease pairs can be represented as:

(15) $$\begin{array}{l} Y_{p}=AB^{T} \end{array}$$

## 4. Results

### 4.1. Experimental Settings and Evaluation

The experiment was performed under 100 trials of five-fold Cross Validation. An average performance was finally computed to reduce the prediction bias. For an MDA matrix ***Y***_*n* × *m*_, CVs were conducted under three different experimental settings as follows.

- Five-fold Cross Validation 1 (CV1): CV on microbes, that is, random rows in ***Y*** (i.e., microbes) were masked for testing.
- Five-fold Cross Validation 2 (CV2): CV on diseases, that is, random columns in ***Y*** (i.e., diseases) were masked for testing.
- Five-fold Cross Validation 3 (CV3): CV on microbe-disease pairs, that is, random entries in ***Y*** (i.e., microbe-disease pairs) were masked for testing.

Under CV1, in each round, 80% of rows in ***Y*** was used as training set and the remaining was used as test set. Under CV2, in each round, 80% of columns in ***Y*** was used as training set and the remaining was used as test set. Under CV3, in each round, 80% of entries in ***Y*** was used as training set and the remaining was used as test set. These three CVs refer to MDA prediction for (1) new (unknown) microbes, (2) new diseases, and new microbe-disease pairs, respectively.

Sensitivity, specificity, accuracy, and AUC were used to evaluate the performances. AUC is the average area under the receiver operating characteristics (ROC) curve. The curve can be plotted by the ratio of True Positive Rate (TPR) to False Positive Rate (FPR) according to different thresholds. TPR and FPR can be computed by Equations (16, 17). High AUC value represents better performance. In our experiments, AUC was computed in each round of CV and final AUC was averaged over the five rounds for 100 times.

(16) $$\begin{array}{l} TPR=\frac{TP}{TP+FN}=\frac{TP}{T} \end{array}$$

(17) $$\begin{array}{l} FPR=\frac{FP}{FP+TN}=\frac{FP}{F} \end{array}$$

where the definitions of *TP*, *FP* and *FN* are as shown in Table 1.

**Table 1:** Confusion matrix of a binary classifier.

	**True class = 1**	**True class = −1**
Predicted class = 1	True positive (TP)	False positive (FP)
Predicted class = −1	False negative (FN)	True negative (TN)

λ is used to determine the probability of jumping between nodes. θ is the restart rate. η denotes the restarting probability in microbe similarity network and disease similarity network. *c* is the importance level of positive samples to negative samples. *K* denotes the number of neighborhood. For the parameters λ, θ, η, *c*, and *K*, we conducted grid search to find the optimal values. RNMFMDA obtained the best performance when these five parameters are set as λ = 0.9, θ = 0.5, η = 0.9, *c* = 8, and *K* = 5. So we set the above five parameters as the corresponding values. Parameters ${\gamma_{m}}^{\prime }$, ${\gamma_{d}}^{\prime }$, and γ are set the same values in previous works, that is, ${\gamma_{m}}^{\prime }=1$, ${\gamma_{d}}^{\prime }=1$, and γ = 0.9. For other parameters, we set the corresponding values according to the method provided by Liu et al. (2016). When ||*P*(*t* + 1) − *P*(*t*)||_*F*_ ≤ 10*e* − 12, the iteration for random walk will stop. The ratio of extracted negative MDAs to positive MDAs is set as 1:1, this is to say, the number of negative MDAs is 450. The parameters in other five methods were set as the same values provided by the corresponding papers.

### 4.2. Performance Comparison of RNMFMDA With Other Five Methods

In this section, we compared our proposed RNMFMDA method with five state-of-the-art MDA prediction models, MDLPHMDA (Qu J. et al., 2019), NGRHMDA (Huang et al., 2017), NTSHMDA (Luo and Long, 2018), LRLSHMDA (Wang et al., 2017), and KATZHMDA (Chen et al., 2017). Tables 2–4 showed the performance of RNMFMDA with other five methods. The best performance is described in boldface in Tables 2–4.

**Table 2:** Performance comparison of RNMFMDA with other five methods under CV1.

**Method**	**Sensitivity**	**Specificity**	**Accuracy**	**AUC**
KATZHMDA	0.2772	0.6690	0.6653	0.3646
LRLSHMDA	0.3286	**0.7538**	**0.7496**	0.4364
NGRHMDA	0.0777	0.3423	0.4817	0.4156
MDLPHMDA	0.3273	0.6890	0.6855	0.4022
NTSHMDA	0.1899	0.6177	0.6138	0.3042
RNMFMDA	**0.4938**	0.6278	0.6274	**0.6332**

*These bolded values represent the best values for the different methods under the same evaluation*.

**Table 3:** Performance comparison of RNMFMDA with other five methods under CV2.

**Method**	**Sensitivity**	**Specificity**	**Accuracy**	**AUC**
KATZHMDA	**0.8317**	0.6487	0.6501	0.8662
LRLSHMDA	0.6944	0.7333	0.7330	0.8086
NGRHMDA	0.3800	0.3285	0.7403	0.8224
MDLPHMDA	0.7318	0.6653	0.6658	0.8178
NTSHMDA	0.7913	0.5905	0.5921	0.8292
RNMFMDA	0.5850	**0.8304**	**0.8283**	**0.8669**

*These bolded values represent the best values for the different methods under the same evaluation*.

**Table 4:** Performance comparison of RNMFMDA with other five methods under CV3.

**Method**	**Sensitivity**	**Specificity**	**Accuracy**	**AUC**
KATZHMDA	**0.8262**	0.6503	0.6518	0.8571
LRLSHMDA	0.7971	0.7412	0.7416	0.8794
NGRHMDA	0.4207	0.3308	0.7796	0.9025
MDLPHMDA	0.8268	0.6729	0.6741	0.8938
NTSHMDA	0.8545	0.5904	0.5926	0.8896
RNMFMDA	0.5810	**0.8818**	**0.8793**	**0.9081**

*These bolded values represent the best values for the different methods under the same evaluation*.

**Figure 1:**
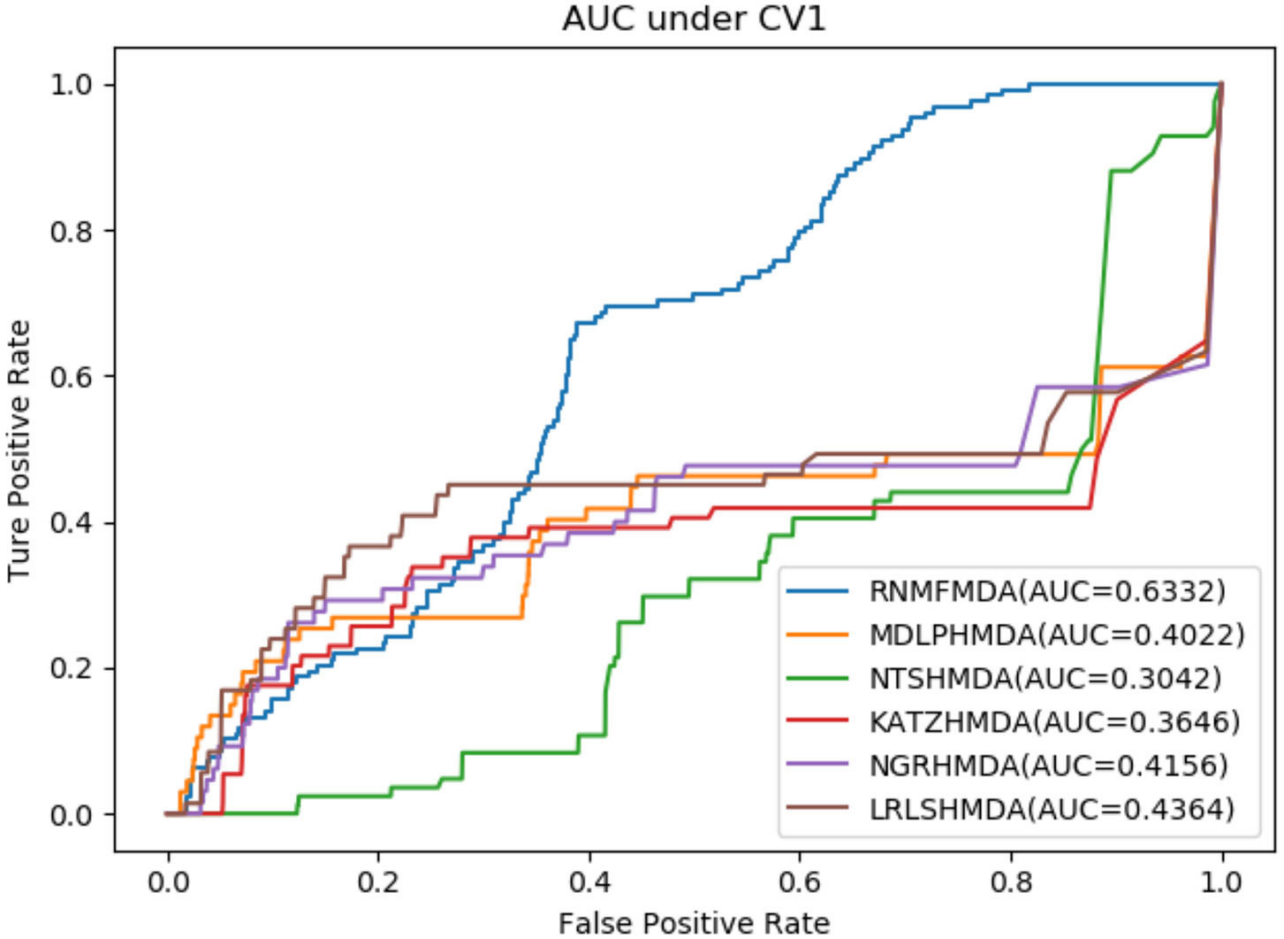
Performance comparison of RNMFMDA with other five methods under CV1.

**Figure 2:**
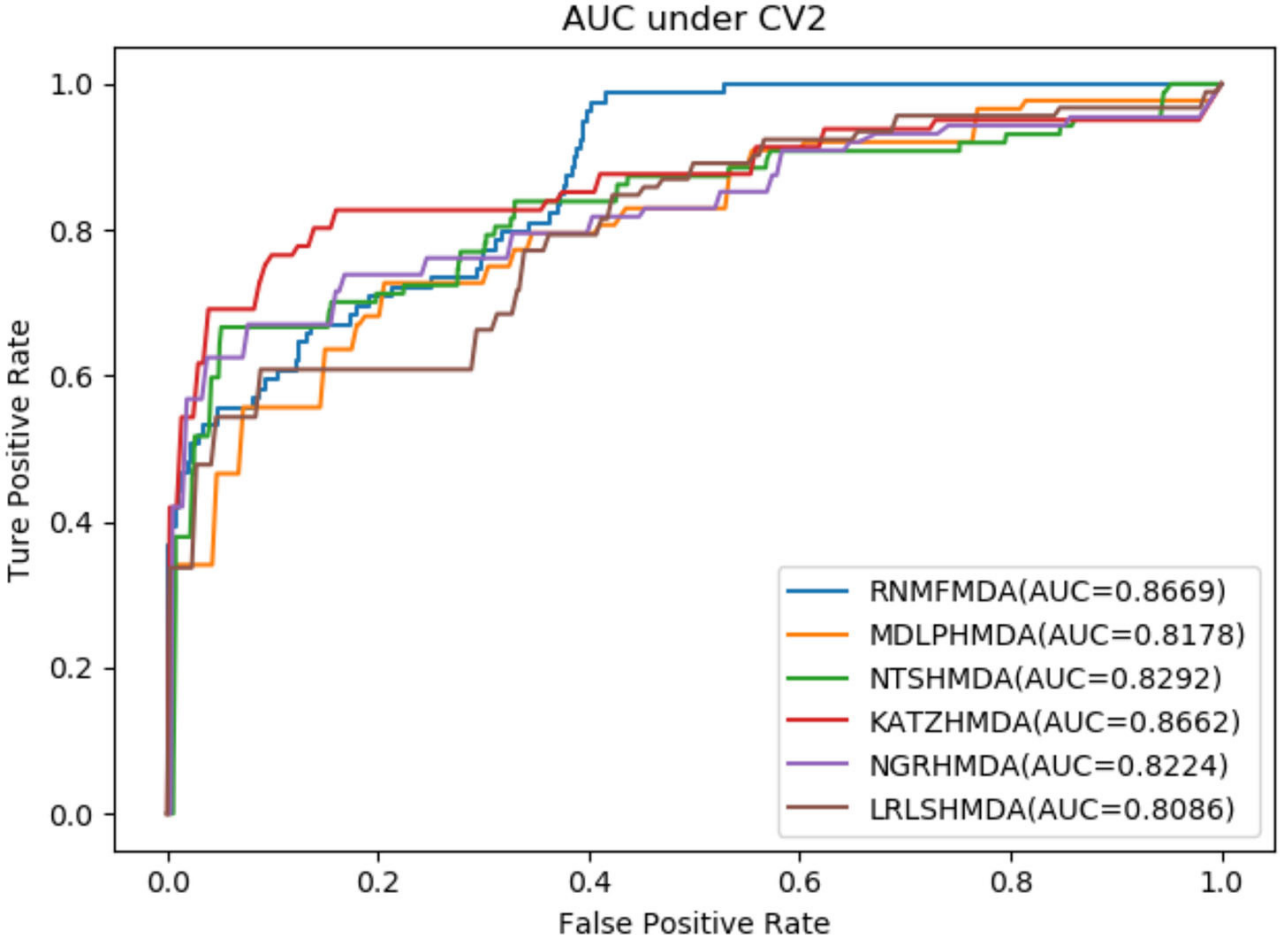
Performance comparison of RNMFMDA with other five methods under CV2.

**Figure 3:**
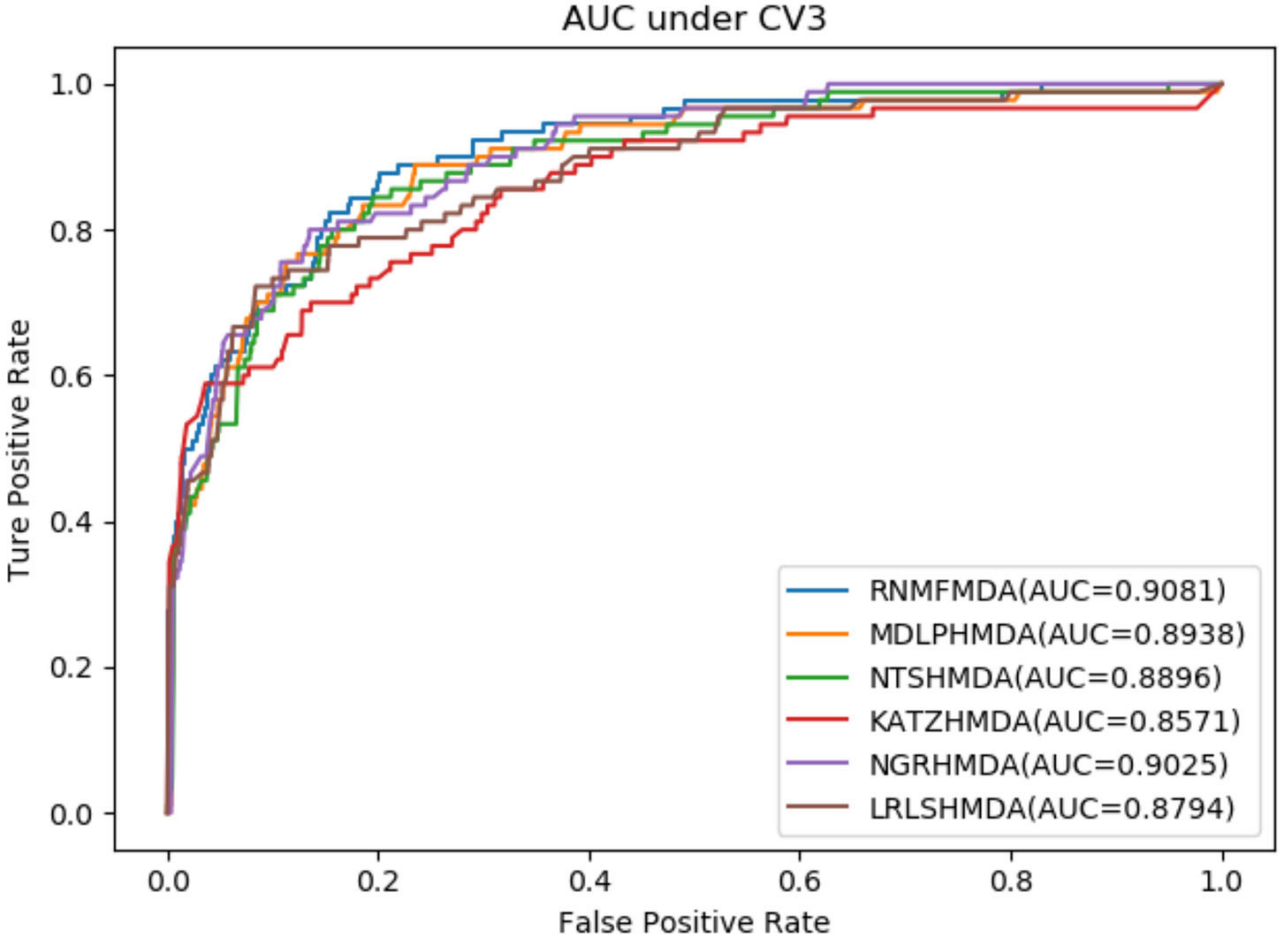
Performance comparison of RNMFMDA with other five methods under CV3.

As shown in Tables 2–4, RNMFMDA performed more efficiently than other five methods. Compared with MDLPHMDA, NGRHMDA, and NTSHMDA, RNMFMDA obtained a more remarkable improvement over four evaluation metrics under three CVs. KATZHMDA and LRLSHMDA are two classic MDA prediction methods. Under CV1, KATZHMDA and LRLSHMDA computed better specificity and accuracy than RNMFMDA. Under CV2 and CV3, these two methods obtained better sensitivity than RNMFMDA. Although KATZHMDA and LRLSHMDA obtained relatively better specificity and accuracy than RNMFMDA under individual CVs, RNMFMDA computed the best AUCs under three CVs. For example, the AUC values in RNMFMDA increased by 42.42, 31.08, 36.48, 34.37, and 51.96% compared with those in KATZHMDA, LRLSHMDA, MDLPHMDA, NGRHMDA, and NTSHMDA under CV1; the corresponding values increased by 0.08, 6.73, 5.66, 5.13, and 4.35%, respectively, under CV2; the values also increased by 5.62, 3.16, 1.57, 0.62, and 2.04%, respectively, under CV3. Figures 1–3 showed the AUCs of these six methods. AUC is a more important measurement compared with other three evaluation metrics. Based on the comprehensive measure of the experimental results, RNMFMDA showed the optimal performance.

In addition, these six methods showed different advantages under different CVs. These variation in improvement can be attributed to differences in data structures under different CVs. In particular, RNMFMDA is more suitable to find possible microbes associated with a given disease.

### 4.3. Performance Comparison Considering PU Learning

In this section, we performed extensive experiments to analyze the influence of different negative MDA selection ratios on prediction performance. Tables 5–7 described the comparison results. NMDAR represents the ratio of selected negative MDA samples to known positive MDA samples.

**Table 5:** Performance comparison considering the number of negative sample CV1.

**NMDAR**	**Sensitivity**	**Specificity**	**Accuracy**	**AUC**
0.0	0.4941	0.5696	0.5696	0.5262
0.1	**0.4945**	0.5933	0.5931	0.5920
0.2	0.4943	0.6131	0.6128	0.6123
0.5	0.4941	**0.6282**	**0.6278**	0.6279
1.0	0.4938	**0.6278**	**0.6274**	**0.6332**
2.0	0.4938	0.6226	0.6223	0.6226
3.0	0.4931	0.6218	0.6216	0.6115
4.0	0.4926	0.6067	0.6066	0.6057
5.0	0.4923	0.5674	0.5676	0.5650

*These bolded values represent the best values for the different methods under the same evaluation*.

**Table 6:** Performance comparison considering the number of negative sample CV2.

**NMDAR**	**Sensitivity**	**Specificity**	**Accuracy**	**AUC**
0	0.5439	0.7621	0.7603	0.7673
0.1	0.5707	0.7676	0.7660	0.8088
0.2	0.6040	0.7678	0.7664	0.8220
0.5	**0.6178**	0.7830	0.7816	0.8410
1.0	0.5850	0.8304	0.8283	0.8669
2.0	0.5581	0.8547	0.8521	0.8791
3.0	0.5560	**0.8575**	**0.8547**	**0.8756**
4.0	0.5492	0.8563	0.8533	0.8782
5.0	0.5461	0.8515	0.8483	0.8734

*These bolded values represent the best values for the different methods under the same evaluation*.

**Table 7:** Performance comparison considering the number of negative sample CV3.

**NMDAR**	**Sensitivity**	**Specificity**	**Accuracy**	**AUC**
0.0	0.5437	0.8559	0.8533	0.8662
0.1	0.5668	0.8565	0.8541	0.8827
0.2	0.6012	0.8532	0.8511	0.8886
0.5	**0.6206**	0.8560	0.8541	0.8970
1.0	0.5810	0.8818	0.8793	0.9081
2.0	0.5612	0.8916	0.8887	**0.9121**
3.0	0.5559	**0.8935**	**0.8904**	0.9096
4.0	0.5527	0.8912	0.8879	0.9099
5.0	0.5459	0.8842	0.8807	0.9026

*These bolded values represent the best values for the different methods under the same evaluation*.

As shown in Tables 5–7, RNMFMDA did not extract negative MDAs when NMDAR is 0, and selected negative MDAs according to different NMDARs of 10, 20, 50%, 1, 2, 3, 4, and 5. When NMDAR is 1, RNMFMDA obtained promising performance under three CVs. Compared with the situation without negative MDA samples, when NMDAR is 1, the AUC values of RNMFMDA respectively increased 16.90, 11.49, and 4.61% under three CVs. Taken as a whole, RNMFMDA with NMDAR of 1 obtained better performance. To reduce overfitting of the experimental results, we selected NMDAR as 1, that is, we extracted negative MDA examples with the same number of positive MDA examples.

**Figure 4:**
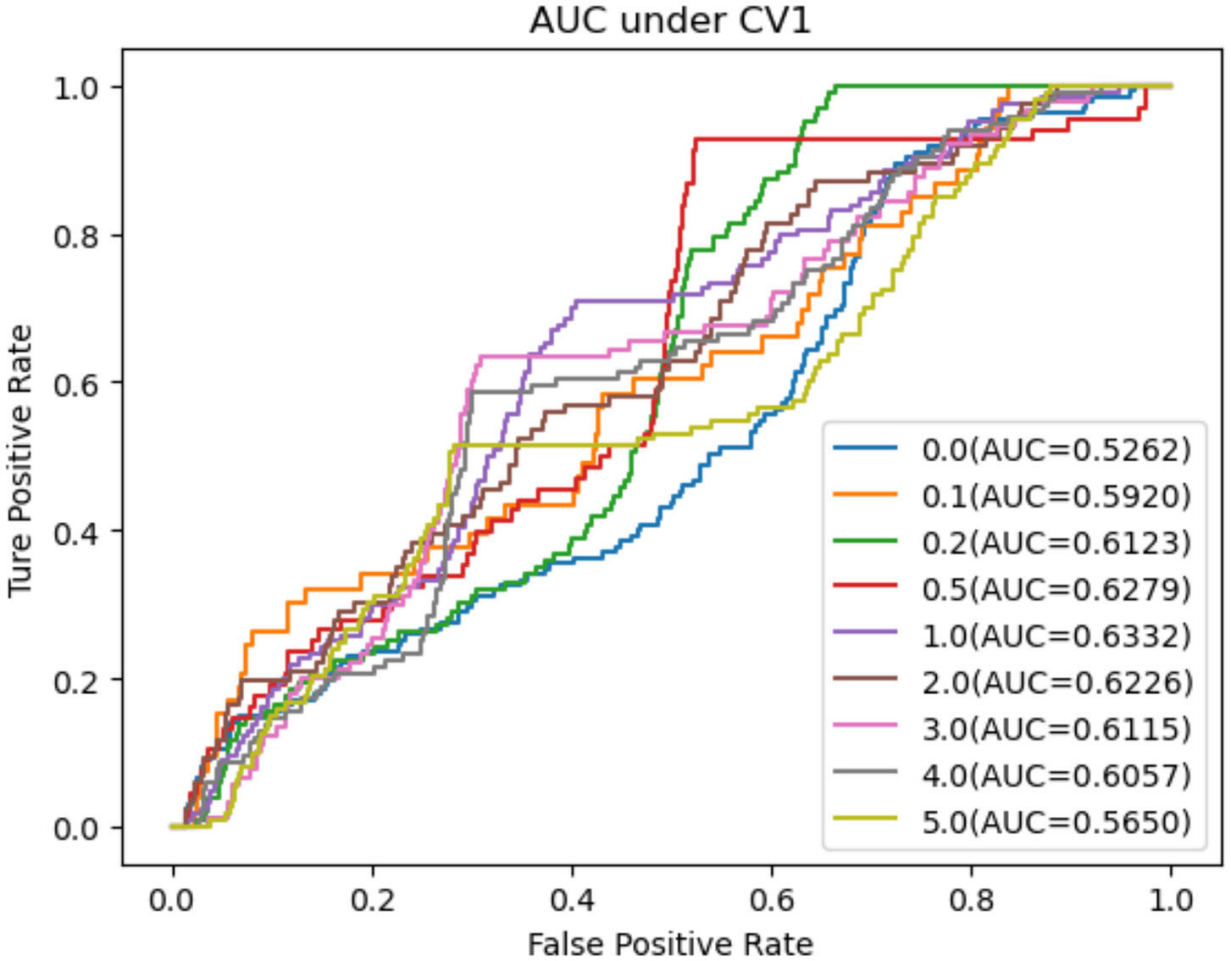
The performance comparison under different negative MDA selection ratios under CV1.

**Figure 5:**
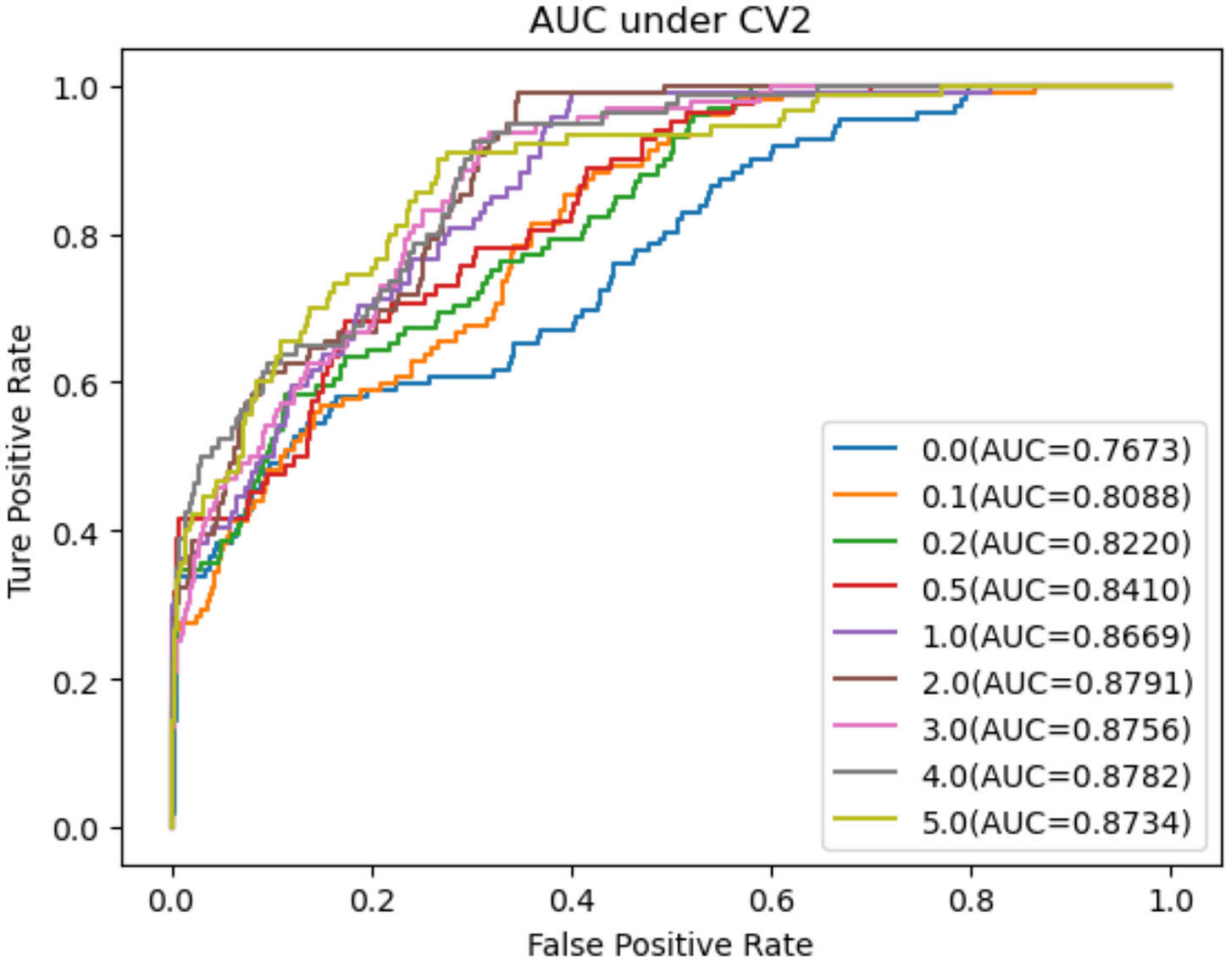
The performance comparison under different negative MDA selection ratios under CV2.

**Figure 6:**
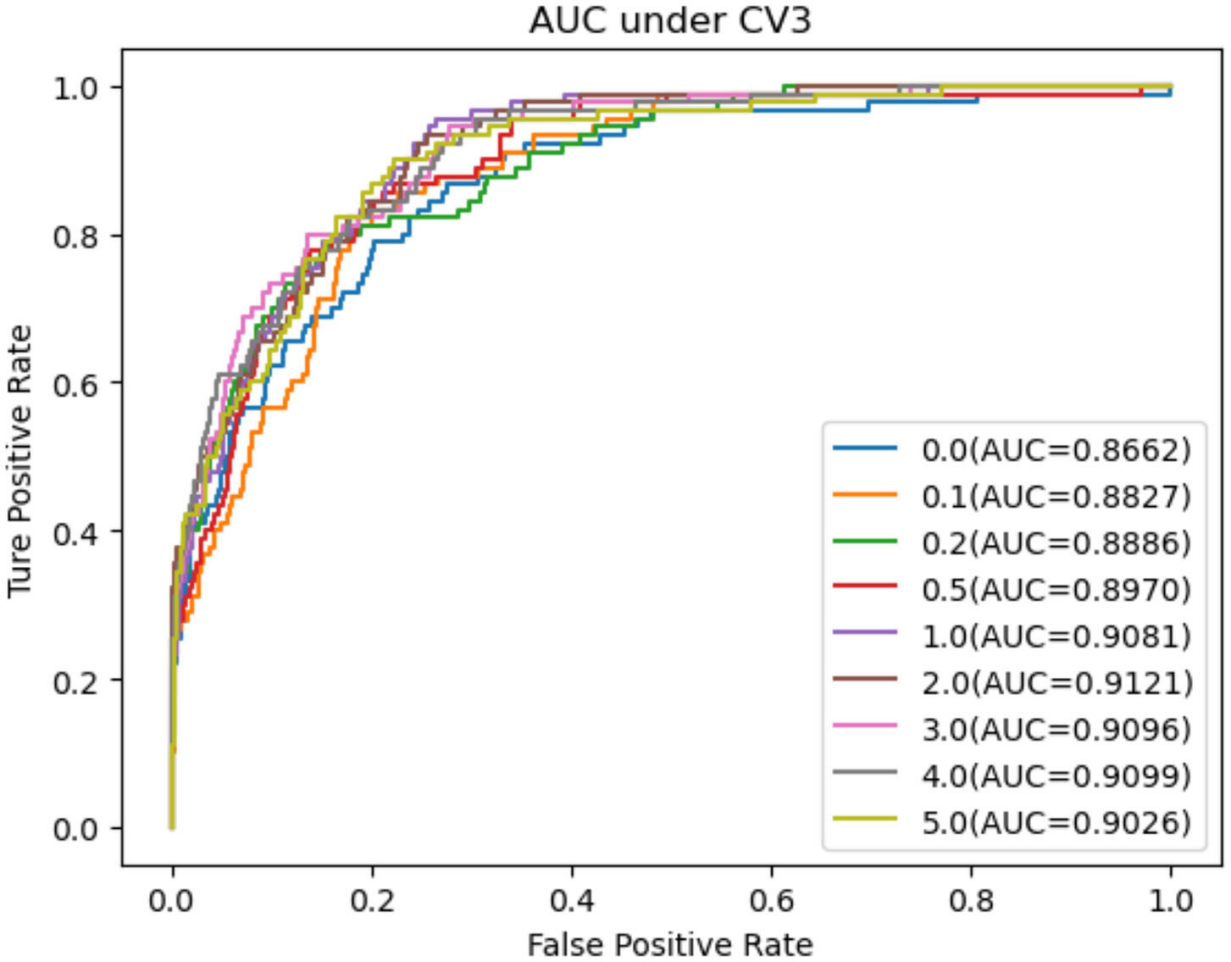
The performance comparison under different negative MDA selection ratios under CV3.

Figures 4–6 showed the AUC values obtained by RNMFMDA under different NMDARs. The results suggested that our proposed negative example extraction method helps to improve MDA prediction.

### 4.4. Case Study

We further evaluated the prediction performance of our proposed RNMFMDA on the confirmed 450 MDAs by two case studies. Asthma is a disease with considerable global morbidity. Over the past 10 years, little improvement in asthma has been observed despite of escalating treatment costs (Pavord et al., 2018). In the first class, we mask all associated information for asthma to find possible microbes. The results are shown in Table 8. Among the predicted top 10 and 20 microbe-asthma association pairs, 8 and 15 microbes have been reported to associate with asthma by related publications, respectively.

**Table 8:** The top 20 microbes associated with asthma.

**Rank**	**Disease**	**Evidence**
1	Clostridium difficile	PMID:21872915
2	Firmicutes	PMID:23265859
3	Bacteroides	PMID:18822123
4	Veillonella	PMID:25329665
5	Clostridia	Unconfirmed
6	Clostridium coccoides	PMID:21477358
7	Actinobacteria	PMID:28947029
8	Collinsella aerofaciens	Unconfirmed
9	Lachnospiraceae	PMID:26220531
10	Lactobacillus	PMID:20592920
11	Enterobacteriaceae	Unconfirmed
12	Staphylococcus aureus	Unconfirmed
13	Streptococcus	PMID:17950502
14	Fusobacterium	DOI:10.4167/jbv.2013.43.4.270
15	Burkholderia	PMID:24451910
16	Enterococcus	PMID:29788027
17	Bifidobacterium	PMID:24735374
18	Klebsiella	PMID:29788027
19	Faecalibacterium prausnitzii	Unconfirmed
20	Pseudomonas	PMID:13268970

Inflammatory Bowel Disease (IBD) is a periodic inflammation. It may be produced by a deregulated immune response to gut microbiome dysbiosis (Halfvarson et al., 2017). In the second class, we mask all association information for IBD to find possible microbes. The results are shown in Table 9. Among the predicted top 10 and 20 microbe-IBD association pairs, there are 9 and 17 microbes that are validated to associate with IBD by recent works, respectively.

**Table 9:** The top 20 microbes associated with asthma.

**Rank**	**Disease**	**Evidence**
1	Helicobacter pylori	PMID:22221289
2	Clostridium difficile	PMID:27698615
3	Bacteroidetes	PMID:25307765
4	Firmicutes	PMID:25307765
5	Prevotella	PMID:25307765
6	Clostridium coccoides	PMID:19235886
7	Bacteroides	PMID:25307765
8	Veillonella	PMID:28842640
9	Clostridia	Unconfirmed
10	Collinsella aerofaciens	PMID:26848182
11	Staphylococcus aureus	PMID:24117882
12	Enterobacteriaceae	Unconfirmed
13	Staphylococcus	PMID:30246806
14	Haemophilus	PMID:24013298
15	Lactobacillus	PMID:26340825
16	Bifidobacterium	Unconfirmed
17	Enterococcus	PMID:24629344
18	Burkholderia	PMID:24325678
19	Streptococcus	PMID:23679203
20	Klebsiella	PMID:29573336

## 5. Discussion

There are numerous microbes in the human body. They play an important role in various biological processes. Many human diseases including gastrointestinal diseases are reported to be closely associated with microorganisms. Therefore, identifying the associations between microbes and diseases helps to understand the pathogenic mechanisms of these diseases and further develop new drugs.

Traditional experimental methods applied to validate possible associations between microbes and diseases are expensive and time-consuming, computational methods are developed to solve with this problem. However, the performance of existing computational models need to further improve. More importantly, lacking of reliable negative MDA examples affects prediction performance. Therefore, RNMFMDA is developed to find possible MDAs.

RNMFMDA obtained the optimal performance under three CVs. We analyzed the reason that RNMFMDA obtained excellent performance and thought that it may be contributed to the following three features. First, we developed a high-quality negative MDA extraction method based on PU learning and random walk with restart. Second, LMFNR is a optimal model in predicting associations between two entities. Finally, we integrated various heterogeneous biological information. Multiple heterogeneous data integration efficiently reflected the biological features of MDAs.

In the future, we will construct a multi-partite network by integrating MDAs, disease-gene associations (Tran et al., 2020), miRNA-disease associations (Peng et al., 2018a; Huang et al., 2019), long non-coding RNA-protein interactions (Zhao et al., 2018; Peng et al., 2019), and long non-coding RNA-disease associations (Chen et al., 2018; Li et al., 2019). More importantly, we will still develop more robust models, for example, ensemble strategy (Hu et al., 2018) and deep learning-based models (Min et al., 2017; Peng L. et al., 2018) to improve MDA prediction.

## Data Availability Statement

All datasets generated for this study are included in the article/Supplementary Material.

## Author Contributions

LP, LS, and LZ: conceptualization. LP: funding acquisition, project administration, writing—original draft, and writing—review and editing. LP and LZ: investigation. LP and LS: methodology. LS, LL, and GL: software. LS: validation. All authors contributed to the article and approved the submitted version.

## Conflict of Interest

The authors declare that the research was conducted in the absence of any commercial or financial relationships that could be construed as a potential conflict of interest.
